# Trigger Thumb, Trigger Finger and Clasped Thumb

**DOI:** 10.3390/children11030294

**Published:** 2024-03-01

**Authors:** Marcos Carvalho, Maria Inês Barreto, Cristina Alves, Francisco Soldado

**Affiliations:** 1 Department of Pediatric Orthopaedics, Pediatric Hospital, Centro Hospitalar e Universitário de Coimbra (CHUC), EPE, Av. Afonso Romão, 3000-602 Coimbra, Portugal; inescbarreto@gmail.com (M.I.B.);; 2 Pediatric Hand Surgery and Microsurgery, Barcelona Children’s Hospital, HM Nens, HM Hospitales, 08009 Barcelona, Spain

**Keywords:** trigger thumb, trigger finger, clasped thumb and thumb in palm

## Abstract

Finger deformities are a common reason for medical observation in children. Subtle clinical differences can have a significant impact on the diagnosis and treatment of these patients. Identification of the basic diagnostic and treatment principles of trigger thumb, trigger finger, and clasped thumb is of paramount importance to all general practitioners, pediatricians, and orthopedic surgeons who are involved in the care of children. The purpose of this article is to review the most important concepts regarding these important topics, focusing on etiology, epidemiology, clinical presentation, diagnosis, treatment and prognosis.

## 1. Introduction

Trigger thumb is one of the most common pediatric hand conditions with a characteristic flexion deformity of the interphalangeal (IP) joint [1]. Another distinct but equally acquired condition is trigger finger, which is far less common than trigger thumb and can be associated with different underlying causes ranging from metabolic diseases to rheumatic pathology [2,3,4]. Clasped thumb, on the other hand, is a congenital deformity of the thumb characterized by a flexion and adduction deformity of the thumb associated with flexion of the IP and metacarpophalangeal (MCP) joints. Unlike trigger thumb, this condition is associated with an abnormal extensor mechanism of the thumb [5,6,7]. To avoid misdiagnosis and excessive and ineffective surgical procedures, clinical suspicion and correct diagnosis are key to appropriate treatment of these thumb deformities. This article aims to review these three relevant conditions, addressing issues related to etiology, epidemiology, diagnosis, treatment and prognosis based on the most recent literature.

### 1.1. Trigger Thumb

#### 1.1.1. Epidemiology, Etiology and Mechanism

Trigger thumb is one of the most common pediatric hand conditions and it is defined as a stenosing tenosynovitis of the flexor pollicis longus (FPL) with a characteristic flexion deformity of the interphalangeal (IP) joint [1]. It affects 1 to 3 per 1000 children and it seems to be more prevalent in Asian and Hispanic populations [3,8,9,10]. The etiology is still controversial but it is probably multifactorial with a possible genetic predisposition [10,11,12], as it occurs more often in monozygotic twins, having an autosomal dominant inheritance pattern with variable penetrance [13,14,15]. Although historically it was often described as congenital, several studies evaluating thousands of newborns have not found any cases of trigger thumbs at birth, despite the known incidence that would lead one to expect its occurrence, supporting the fact that it is an acquired condition [9,11,12]. Unlike trigger finger in adults, histological studies using electron microscopic analysis of longitudinal sections of the A1 pulley and Notta’s nodule in pediatric age revealed large amounts of mature collagen and fibroblasts but no degenerative or inflammatory changes [16]. Although debated, one of the most widespread etiologic theories is that of microtrauma [8]. It is theorized that the naturally flexed and adducted position of the neonatal thumb, associated with the strength of the grasp reflex, may be the cause of subtle trauma to the FPL. This process results from an anatomical mismatch between the diameter of the FPL and the A1 pulley, which would lead to tendon swelling and/or pulley thickening [8]. In addition to the classically described A1 pulley, there is also a potential contribution from another pulley, the variable pulley (Av), which could lead to additional stenosis of the FPL tendon [17,18,19].

#### 1.1.2. Clinical Presentation and Diagnosis

**Figure 1:**
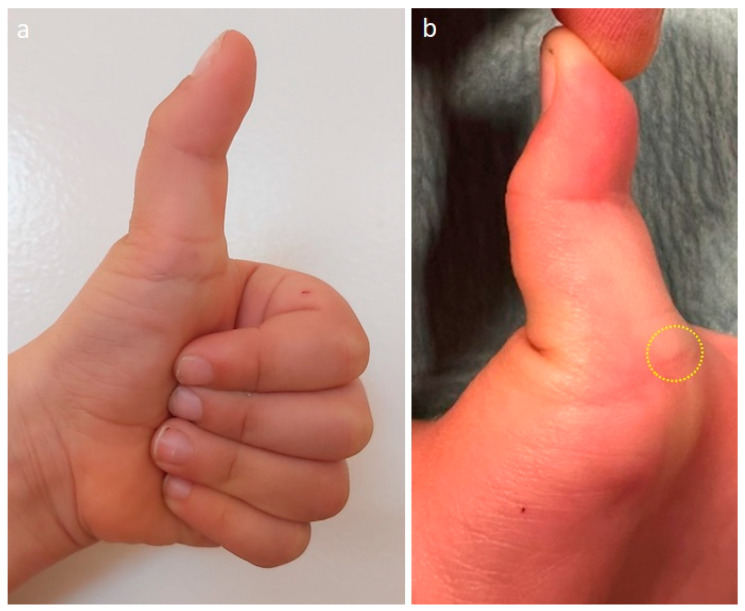
(**a**) Left trigger thumb with typical clinical appearance with the thumb locked in flexion at the IP joint; (**b**) compensatory hyperextension of the MCP joint is visible when the IP joint of the thumb is extended. The yellow circle identifies Notta’s nodule at the level of the MCP volar crease.

Most patients with trigger thumb are brought to the attention of a physician around the age of 2 [11,20,21]. The patient usually presents with one or both thumbs locked in flexion at the IP joint (Figure 1). This deformity is painless in this position, but commonly the patient will experience some discomfort when the thumb IP joint is forcefully extended, resulting in hyperextension of the MCP joint (Figure 1) [3]. Another common finding is a painless palpable nodule on the volar aspect of the MCP joint flexion crease called Notta’s nodule. It is rare to observe a “real triggering” in the thumb as we often see in the other fingers. Nevertheless, it may be present and is reported by parents as occurring very occasionally at home and is rarely noticeable or reproducible in the office at the time of the consultation. There are two well-known classifications, Sugimoto and Watanabe, based mainly on the presence of active triggering, passive triggering (Video S1—Sugimoto’s stage III; Watanabe’s: stage 2) or rigid deformity in the most severe stage (Table 1) [22,23]. The diagnosis of this pathology is purely clinical and there is no need for any diagnostic tests. The differential diagnosis should include the congenital clasped thumb, especially Tsuyuguchi type I—where we have a supple clasped thumb with isolated weakness of the extrinsic extensor tendons [24]. Other differential diagnoses to consider apart from partial or complete absence of the extensor apparatus include arthrogryposis, dislocation or fracture of the thumb or flexor tendon entrapment in the metacarpal head after trauma [15].

#### 1.1.3. Treatment

**Figure 2:**
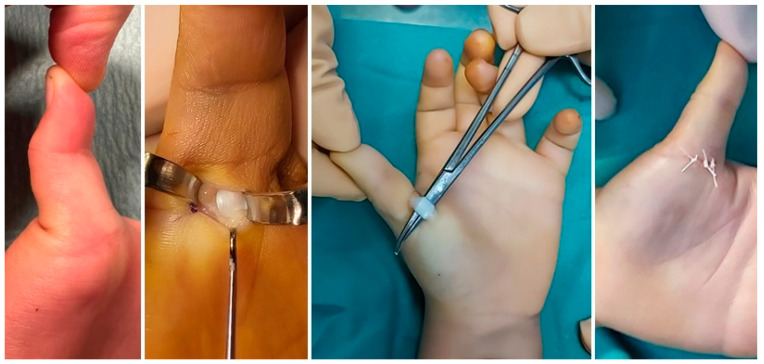
Intra-operative photographs of the open surgical treatment of the trigger thumb of a 3-year-old boy, with sectioning of the A1 pulley and release of the flexor pollicis longus tendon, allowing correction of the deformity and full extension of the interphalangeal joint.

Regarding the treatment of trigger thumb, there is no consensus on the initial approach, the therapeutic options or the best time for treatment [25]. Treatment options range from observation (no intervention) to conservative treatment (exercise and/or splints) and to surgical treatment (open or percutaneous) [1,26,27,28,29,30,31,32]. It is recognized that many cases resolve spontaneously, although the results in the literature vary even when considering similar follow-up times [1,26]. For this reason, a period of observation is acceptable in the expectation of self-limited resolution, although it is known that patients with rigid flexion of the IP joint of more than 30° have a very low rate of resolution, making the indication for surgery even more justified [26]. Conservative treatment with exercises or splints is an option, particularly used in Asian countries, with success rates ranging from 32 to 92% [33,34,35]. Successful treatment depends on the compliance of the patient and family, and the literature suggests that the more passively rigid the deformity is, the lower the success rate [33,34,35]. Of the several options available, the use of steroid injection or percutaneous release of the A1 pulley do not have the same safety profile or scientific acceptance and are therefore not techniques commonly recommended in pediatric patients [30,31,32]. Percutaneous release is associated with a significantly higher risk of recurrence, iatrogenic neurovascular or FPL injuries than open surgery, and the literature suggests that open surgery provides the most reliable results [30,31,32]. This is also the approach used by the first and third authors of this article, who believe that open surgery is the best treatment method for trigger thumb (Figure 2). In cases of thumb deformity with passively reducible flexion of the IP joint, the authors wait 6 months for self-limiting resolution, and if this does not happen, surgery is recommended. In cases of rigid IP joint flexion over the age of 2, we recommend surgery. We do not perform this surgery under the age of 2.

#### 1.1.4. Prognosis

Trigger thumb has a good prognosis, with about 32–76% of trigger thumbs resolving their condition spontaneously with complete resolution after 5 years of follow-up without the need for any kind of intervention [1,26]. However, it is known that conservative and surgical treatment are associated with higher rates of resolution, with open surgery yielding the best results [29,30,31,32]. The outcome of conservative treatment depends not only on the specificity of the treatment method and the initial severity of the deformity, but also on the patient’s and family’s compliance, which is why there is a wide variability in results, as described previously [33,34,35]. Open surgical treatment is associated with excellent results and lower rates of recurrence or iatrogenic neurovascular injury compared to the percutaneous technique, although it is not exempt from complications such as incomplete release, wound complications, bowstringing, nerve injury, IP flexion deficit, and metacarpophalangeal joint hyperextension [1,27,29].

### 1.2. Trigger Finger

#### 1.2.1. Epidemiology, Etiology and Mechanism

The trigger finger is a separate entity from the trigger thumb, and its occurrence is up to 10 times less common than in the thumb [36]. The etiology seems to be related to various factors, from trauma, anatomical anomalies or other associated diseases such as mucopolysaccharidosis or diabetes [2,3]. The presence of a trigger finger should also alert us to the possibility of rheumatoid disease (juvenile idiopathic arthritis) or collagen diseases, so it is important to keep these entities in mind as pathophysiologically favoring this condition. In terms of etiology, trigger finger is very different from its adult counterpart, arising due to a different set of anatomical conditions, such as the presence of anatomical abnormalities, including the thickening/nodularity of the flexor digitorum superficialis (FDS) or flexor digitorum profundus (FDP) tendons, slips or adhesions between the FDS and the FPL, proximal FDS decussation, abnormal slips of the FDS or constriction of A1, A2 or A3 pulleys [2,3]. According to Wong’s systematic review [2], the majority of these patients (54%) have only unilateral and single finger disease and the most commonly involved fingers are the 3th (44%) and the 4th (30%). The same article mentions that 29% of these patients have an underlying condition that predisposes them to the disease, most commonly mucopolysaccharidosis and related lysosomal storage diseases [2]. Patients with trigger finger associated with underlying systemic conditions often have bilateral or multiple digit involvement or concomitant carpal tunnel syndrome [2].

#### 1.2.2. Clinical Presentation and Diagnosis

**Figure 3:**
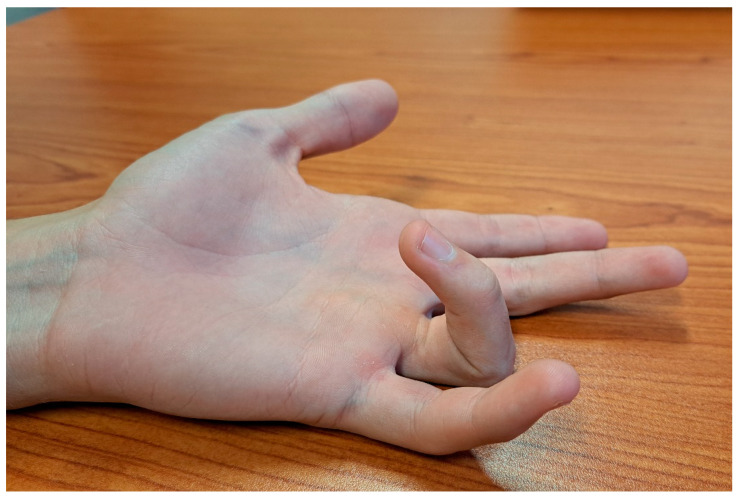
Clinical photograph of a 15-year-old patient with no history of underlying pathology with a fourth trigger finger, illustrating the moment before the finger snaps, which occurs when the patient is asked to extend all fingers. (In addition, Video S2 makes it possible to perceive the entire movement of the finger, from the flexion to the appearance of the trigger and to the full extension).

Most cases of trigger finger occur in the first 4 years of life, but it can also occur later, between the ages of 10 and 12 [36,37,38]. This condition most commonly presents as snapping and triggering of the finger, while less commonly, a fixed flexion deformity such as trigger thumb is seen. In some patients, it is also possible to identify a volar mass at the level of the MCP joint, representing a nodularity of the FDS or FDP due to tightening and continuous tendon friction caused by the A1 pulley during flexion and extension of the finger (Figure 3, Video S2) [3,36,37,38]. There may also be a painless click with digital manipulation, and in some cases, patients may report decreased range of motion, flexed finger posture, or secondary contractures of the PIP joint [3]. These patients may have one or more affected fingers, so in the clinical assessment, it is important to observe and test all the digits of the hand [3]. In addition, we should try to clinically understand the specific location of the trigger, as this will help us to perceive the anatomical site responsible for the discomfort, thus facilitating perception for the purposes of the subsequent surgical therapeutic approach. As with trigger thumb, the diagnosis is clinical and there is no need for imaging studies. However, it should be noted that children with this condition may have other underlying pathologies, so the diagnosis should consider the exclusion of these disorders [2]. The diagnostic work-up should therefore be directed toward investigating the underlying causes that may be responsible for the trigger finger. For this reason, radiographs may be useful to exclude bone mass, ultrasound may be used to evaluate flexor tendon nodules, inflammatory processes, or pulley thickening, and MRI can be used to evaluate soft tissue lesions, although it rarely changes the therapeutic approach and has the disadvantage of requiring sedation in small children [2]. If mucopolysaccharidosis is suspected, urine should be screened for glycosaminoglycans, and if positive, these patients should be referred for genetic evaluation [2]. Differential diagnosis should include congenital extensor mechanism deficiency, camptodactily and lateral band snapping syndrome [3,39].

#### 1.2.3. Treatment

**Figure 4:**
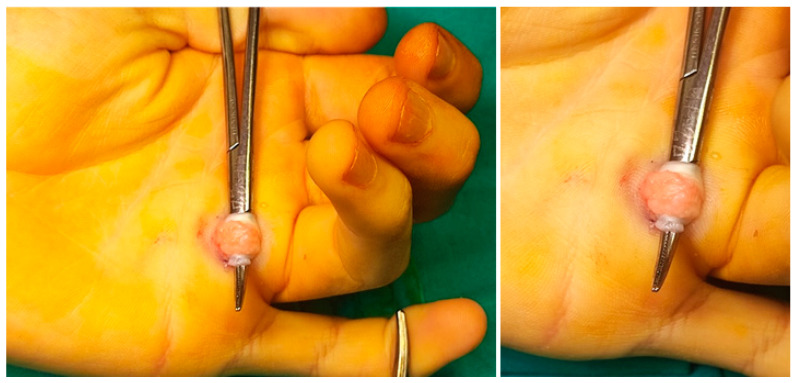
Intraoperative photographs of an open surgical treatment of a patient with no history of underlying systemic disease with a fourth trigger finger caused by an atypical nodule of the tendon and extensive local inflammation. In this case, the A1 pulley was sectioned and, due to the mechanical conflict caused by the nodule and the local inflammatory phenomenon, the local peritendinous tissue had to be excised, allowing intra-operative resolution of the trigger and subsequent clinical resolution, without recurrence.

Regarding the treatment of trigger finger, an attempt at conservative treatment seems to be acceptable if there is no suspicion of metabolic disease or underlying anatomical abnormalities [2]. Nevertheless, approximately one third of these idiopathic cases do not resolve and end up requiring surgery [2]. If surgery is being considered, the procedure should begin with division of the A1 pulley and assessment of flexor excursion following this release. Unlike adults, this surgical step is often not sufficient to release the trigger and achieve full extension of the digit in children. If this is the case, the surgeon should look for other potential causes of mechanical conflict, such as nodules, abnormal FDS slips, abnormal lumbrical insertions, or adhesions between the FDS and FDP (Figure 4) [2,4]. If abnormal masses or structures are identified, they should be excised. If the trigger finger remains, it may be necessary to resect one or both FDS slips and/or partially resect the A2 pulley or resect the A3 pulley [2,4]. In the presence of trigger finger in a patient with mucopolysaccharidosis, it seems acceptable to try conservative treatment, but if there is no resolution within 6 months, surgical treatment should be pursued. In these patients, the initial approach is more aggressive, opting for sectioning of the A1 and A3 pulleys, sectioning of an FDS slip and release of the carpal tunnel concurrently [2,4]. In older patients with the suitable profile, it is possible to perform this procedure using the wide-awake local anesthesia no-tourniquet technique (WALANT), which can be useful in a step-wise approach, allowing us to sequentially understand the causes that contribute to the triggering of the finger, which can be actively extended by the patient during the procedure [40].

#### 1.2.4. Prognosis

Trigger finger is associated with a good prognosis, with open surgery being associated with high success rates and low complication and recurrence rates [2,4]. Despite the fact that many of them occur in patients with concomitant systemic/metabolic pathology, the literature indicates that even in such patients, recurrence rates after surgery are low (6%), although it is important to highlight the short follow-up of patients in these studies [2]. It should be noted, however, that the therapeutic approach to trigger finger varies depending on the etiology and is more aggressive in the presence of an underlying medical condition [2,4].

### 1.3. Clasped Thumb

#### 1.3.1. Epidemiology, Etiology and Mechanism

Congenital clasped thumb is a deformity of the thumb characterized by a flexion-adduction deformity of the thumb associated with flexion of the IP and MCP joints [5,6]. Clasped thumb is often bilateral, male predominant and is associated with an abnormal thumb extensor mechanism (extensor pollicis brevis (EPB), longus (EPL) or both) that prevents the thumb from being actively extended [5,6,7]. This deformity is often associated with other musculoskeletal diseases and approximately 68% are associated with various syndromes [7] (Freeman–Sheldon, Emery–Nelson, Moebius, Waardenburg, congenital contractural arachnodactyly, digitotalar dysmorphism, arthrogryposis and multiple pterygium) [5,6,7,41,42]. In these syndromic patients, it is common to see other congenital flexion finger deformities [5,6,7,41,42]. At birth, the thumb is in the palm, which is a normal and physiological position for the first 3 to 6 months of life. This flexed posture is a consequence of the intrauterine position of the wrist and fingers with the fingers in a fist position in which the other fingers overlap the thumb. With birth, it is expected that the thumb can be easily mobilized passively in extension and that progressively, this posture will resolve spontaneously, with normal function of the extensor mechanism [5,6,7,20,41,42]. If this evolution does not occur, the patient may have a congenital anatomical malformation that prevents this movement. In addition to clasped thumb deformity, these patients may concomitantly have syndactyly of digital web spaces, collateral ligament tightness, palmar plate contracture and hypoplasia of joint surfaces, affecting congruence and normal joint mobility and stability [5,6,7].

#### 1.3.2. Clinical Presentation and Diagnosis

**Figure 5:**
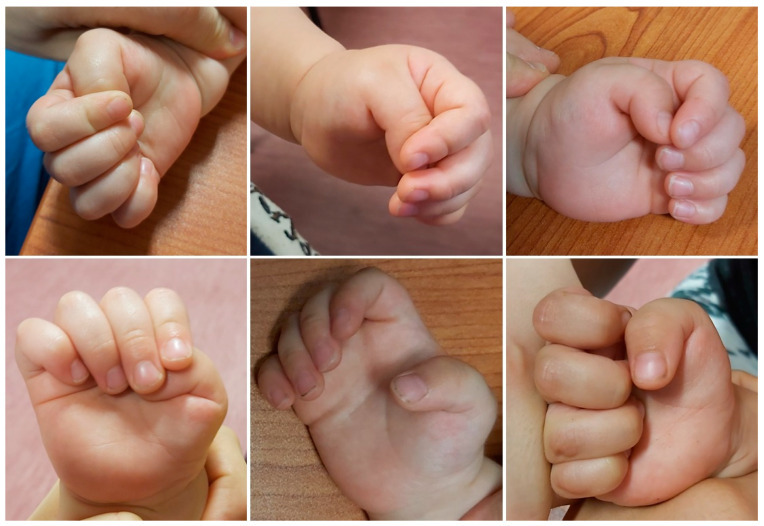
Clinical photographs of patients with clasped thumbs of varying severity, showing flexion and adduction thumb deformity with a fulcrum at the metacarpophalangeal joint.

Patients with congenital clasped thumb present clinically with a flexion and adduction deformity of the thumb, which is usually positioned in the palm underneath the other four digits (Figure 5) [5]. Although the thumb-in-palm posture is physiologic up to 6 months of age, it is expected that the thumb will be easily passively extended and that active extension will be present after this period. In patients with a clasped thumb, this does not happen and there is either continued weakness or a complete lack of thumb extension, leaving a thumb in palm deformity with an MCP joint fulcrum (Figure 5) [5,7,43]. In the most severe cases, with complete absence of the EPL and EPB, the thumb crosses almost the entire palm and lies at the level of the base of the 5th finger [5]. This deformity is also often associated with other secondary deficiencies such as first web space narrowing and contracture, collateral ligament tightness, palmar plate contracture, and changes in joint congruency [5]. According to the severity of the deformity, Tsuyugushi classified these deformities into the following three types: Type I—supple clasped thumb (the thumb could be passively abducted and extended against the resistance of thumb flexor, without other digital anomalies); Type II—clasped thumb with hand contractures (the thumb could not be passively extended and abducted, with or without other digital anomalies); Type III—clasped thumb associated with arthrogryposis [5,24].

#### 1.3.3. Treatment

**Figure 6:**
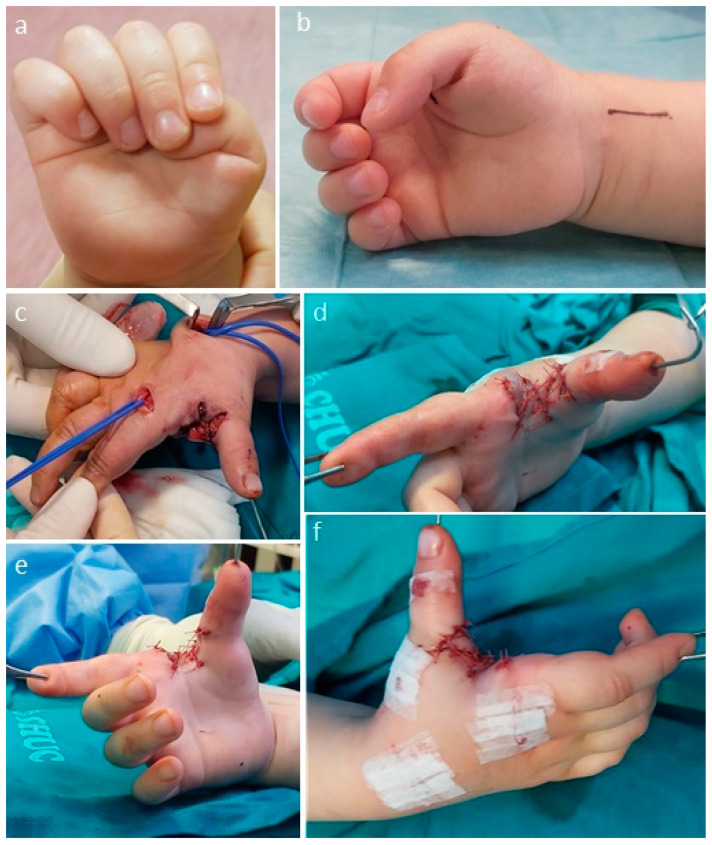
Intra-operative photographs of the surgical treatment of a clasped thumb (Tsuyugushi Type I). An attempt at conservative treatment with a thumb extension splint was unsuccessful and led to an indication for surgical treatment. (**a**,**b**) Preoperative photographs. (**c**,**d**) A four-flap Z-plasty and release of the intrinsic tenar muscles was performed to widen the first web space, and an MCP joint stabilization (double breasting of the capsule and K-wire fixation) and a tendon transfer of the extensor indicis were also performed. (**e**,**f**) Immediate postoperative photographs to illustrate the correction achieved.

Regarding the treatment of clasped thumb, there are conservative and surgical options [5,6,7,44]. Conservative treatment is usually reserved for younger patients, ideally under 1 year of age, who present flexible deformities that allow passive correction [7]. In these cases, although it varies, protocols have been described that include wearing a splint full time with the thumb in extension for approximately 6 months and then, after achieving full extension of the thumb, moving to wearing the splint only at night for another 6 months [7]. If this treatment protocol is ineffective, surgical treatment may offer the possibility of correcting the deformity [7]. Surgical treatment varies according to the degree of deformity and should focus on correcting the various components that may contribute to the overall deformity of the thumb [6,7,44]. Therefore, it may be necessary to open the first interdigital space with skin flaps (Z-plasty, four-flap Z-plasty, stiletto flap or dorsal rotation advancement flap), release the fascia and carpometacarpal capsule, and release the intrinsic muscles (adductor pollicis and first dorsal interosseus) (Figure 6) [6,7,44]. It is also critical to stabilize the MCP joint (capsule plication, ligament reconstruction and/or joint transfixion with K-wire, chondrodesis) and to rebalance the musculature (extensor indicis or flexor digitorum superficialis tendon transfer and FPL lengthening) (Figure 6) [6,7,44].

#### 1.3.4. Prognosis

The prognosis for clasped thumb varies depending on the severity of the deformity. In mild deformities that are sufficiently flexible to undergo passive mobilization, or with only extensor tendon weakness, conservative treatment (splinting) provides good results. However, in cases of deformities with late presentation (after 2 years old), marked first web space/hand contractures without passive extension of the thumb (Tsuyugushi’s Type II) or when associated with arthrogryposis (Tsuyugushi Type III), its effectiveness is limited and surgical treatment remains the most reliable option [6,7,44].

## 2. Conclusions

The purpose and importance of this article is to provide a global approach to these three relevant pathologies in pediatric hands (Table 2). The relevance of recognizing and differentiating between a trigger thumb and a clasped thumb allows for proper management and avoidance of inappropriate surgical procedures or therapeutic approaches that may be harmful to the patient. Another important message is that trigger fingers in children, other than the thumb, are very uncommon and may be associated with underlying systemic conditions that should be excluded (rheumatic, endocrinologic, or metabolic conditions). Awareness of these concepts should be embraced by all general practitioners, pediatricians and orthopedic surgeons, allowing for early referral and timely observation of these patients in specialized units whenever clinically warranted, allowing for proper counseling of parents and the best treatment for their children.

## Figures and Tables

**Table 1:** Trigger thumb classifications according to *Sugimoto* and *Watanabe*.

Sugimoto’s Classification [22]	
Stage I	Palpable Notta’s nodule without passive or active triggering
Stage II	Triggering occurs with an active extension of the thumb IP joint
Stage III	The thumb cannot be actively extended and triggering occurs with passive extension
Stage IV	The IP joint cannot be extended passively; fixed flexion deformity exists
Watanabe’s Classification [23]	
Stage 0	Normal
Stage 1	Locking in flexion or extension/active movement with a trigger
Stage 2	Locking in flexion or extension/Passive movement with a trigger
Stage 3	Locking in flexion or extension

**Table 2:** Trigger thumb, trigger finger, and clasped thumb: key clinical features, diagnostic pearls, and best treatment option.

	Key Clinical Features	Diagnostic Pearls	Best Treatment Option
Trigger Thumb	- Thumb locked in flexion at the IP joint - Hyperextension of the MCP joint - Notta’s nodule	- Clinical diagnosis (no diagnostic tests are required) - Do not confuse it with the clasped thumb. Look at the MCP joint ؞ MCP joint is not flexed ؞ Active extension of the MCP joint is preserved	Open Surgery
Trigger Finger	- Snapping and triggering of the finger - Painless clicking with digital manipulation	- Can affect multiple fingers—evaluate both hands - Underlying disease in 29% ؞ MPS, diabetes, JIA ؞ If MPS is suspected, urine should be screened for GAG, and if positive, referred for genetic evaluation	Open Surgery
Clasped Thumb	- Flexion-adduction deformity of the thumb with a fulcrum at MCP joint - Abnormal thumb extensor mechanism - Possible association with other hand disorders (syndactyly, camptodactyly, joint stiffness or ligamentous instability)	- Associated syndromes in 68% of patients ؞ Freeman–Sheldon, Emery–Nelson, Moebius, Waardenburg, congenital contractural arachnodactyly, digitotalar dysmorphism, arthrogryposis, multiple pterygium	Mild Deformity ؞ Splinting Moderate/Severe Deformity ؞ Surgery

GAG: glycosamynoglycans; IP: interphalangeal; JIA: juvenile idiopathic arthritis; MCP: metacarpophalangeal; MPS: mucopolysaccharidosis.

## Data Availability

Not applicable.

## Supplementary Materials

The following supporting information can be downloaded at: https://www.mdpi.com/article/10.3390/children11030294/s1, Video S1: trigger thumb; Video S2: trigger finger; Video S3: trigger finger surgery.

## References

[1] Baek G.H., Lee H.J. (2011). The natural history of pediatric trigger thumb: A study with a minimum of five years follow-up. Clin. Orthop. Surg..

[2] Wong A.L., Wong M.J., Parker R., Wheelock M.E. (2022). Presentation and aetiology of paediatric trigger finger: A systematic review. J. Hand Surg..

[3] Shah A.S., Bae D.S. (2012). Management of pediatric trigger thumb and triggerfinger. J. Am. Acad. Orthop. Surg..

[4] Womack M.E., Ryan J.C., Shillingford-Cole V., Speicher S., Hogue G.D. (2018). Treatment of paediatric trigger finger: A systematic review and treatment algorithm. J. Child. Orthop..

[5] Rayan G.M., Upton J. (2014). Congenital Hand Anomalies and Associated Syndromes.

[6] Kim S., Kwak W.K., Jung S.T. (2021). Characteristics of Congenital Clasped Thumb: A Case Report and Literature Review. Front. Pediatr..

[7] Ghani H.A., El-Naggar A., Hegazy M., Hanna A., Tarraf Y., Temtamy S. (2007). Characteristics of patients with congenital clasped thumb: A prospective study of 40 patients with the results of treatment. J. Child. Orthop..

[8] Bauer A., Bae D. (2015). Pediatric trigger digits. J. Hand Surg..

[9] Kikuchi N., Ogino T. (2006). Incidence and development of trigger thumb in children. J. Hand Surg..

[10] Ashford J.S., Bidic S.M. (2009). Evaluation of pediatric trigger thumb in the hispanic population at a southwest urban medical center. Plast. Reconstr. Surg..

[11] Slakey J.B., Hennrikus W.L. (1996). Acquired thumb flexion contracture in children: Congenital trigger thumb. J. Bone Jt. Surg. Br. Vol..

[12] Rodgers W.B., Waters P.M. (1994). Incidence of trigger digits in newborns. J. Hand Surg..

[13] Shim V.C., Admire A.A., Heidenreich R.A., Samimi K.J. (2002). Autosomal dominant inheritance pattern for trigger thumb. Plast. Reconstr. Surg..

[14] Wang E.D., Xu X., Dagum A.B. (2012). Mirror-image trigger thumb in dichorionic identical twins. Orthopedics.

[15] Ahmadi S., Akbari H., Shafaei Y., Akbari P. (2021). Trigger Thumb in Twins: Case Report. World J. Plast. Surg..

[16] Buchman M.T., Gibson T.W., McCallum D., Cuda D.D., Ramos A.G. (1999). Transmission electron microscopic pathoanatomy of congenital trigger thumb. J. Pediatr. Orthop..

[17] Doyle J.R., Blythe W.F. (1977). Anatomy of the flexor tendon sheath and pulleys of the thumb. J. Hand Surg..

[18] Bayat A., Shaaban H., Giakas G., Lees V. (2002). The pulley system of the thumb: Anatomic and biomechanical study. J. Hand Surg..

[19] Schubert M.F., Shah V.S., Craig C.L., Zeller J.L. (2012). Varied Anatomy of the Thumb Pulley System: Implications for Successful Trigger Thumb Release. J. Hand Surg..

[20] Dinham J.M., Meggitt B.F. (1974). Trigger thumbs in children: A review of the natural history and indications for treatment in 105 patients. J. Bone Jt. Surg. Br. Vol..

[21] Marek D.J., Fitoussi F., Bohn D.C., Van Heest A.E. (2011). Surgical release of the pediatric trigger thumb. J. Hand Surg..

[22] Sugimoto Y. (1991). Treatment of trigger digit in children. Seikei Geka.

[23] Watanabe H., Hamada Y., Toshima T., Nagasawa K. (2001). Conservative treatment for trigger thumb in children. Arch. Orthop. Trauma. Surg..

[24] Tsuyuguchi Y., Masada K., Kawabata H., Kawai H., Ono K. (1985). Congenital clasped thumb: A review of forty-three cases. J. Hand Surg..

[25] Carvalho M., Perez-Lopez L., Farr S., Catena N. (2023). Trigger thumb treatment approach: Results of a survey of EPOS members. J. Child. Orthop..

[26] Hutchinson D.T., Rane A.A., Montanez A. (2021). The natural history of pediatric trigger thumb in the United States. J. Hand Surg..

[27] Dittmer A.J., Grothaus O., Muchow R., Riley S. (2020). Pulling the trigger: Recommendations for surgical care of the pediatric trigger thumb. J. Pediatr. Orthop..

[28] Yano K., Ikeda M., Yoneda M., Tokui A., Nakagawa K., Kaneshiro Y., Hosomi R., Kazuki K. (2021). Clinical results of splinting versus observation for pediatric trigger thumb. J. Pediatr. Orthop. B.

[29] Farr S., Grill F., Ganger R., Girsch W. (2014). Open surgery versus nonoperative treatments for paediatric trigger thumb: A systematic review. J. Hand Surg..

[30] Yan L., Jiang L., Qu X., Liu X., Li M., Wu J. (2021). Efficacy analysis of day surgery A1 pulley release for pediatric trigger thumb. Front. Pediatr..

[31] Sirithiantong T., Woratanarat P., Woratanarat T., Angsanuntsukh C., Saisongcroh T., Unwanatham N., Thakkinstian A. (2021). Network meta-analysis of management of trigger thumb in children. J. Pediatr. Orthop. B.

[32] Masquijo J.J., Ferreyra A., Lanfranchi L., Torres-Gomez A., Allende V. (2014). Percutaneous trigger thumb release in children: Neither effective nor safe. J. Pediatr. Orthop..

[33] Koh S., Horii E., Hattori T., Hiroishi M., Otsuka J. (2012). Pediatric trigger thumb with locked interphalangeal joint: Can observation or splinting be a treatment option?. J. Pediatr. Orthop..

[34] Nemoto K., Nemoto T., Terada N., Amako M., Kawaguchi M. (1996). Splint therapy for trigger thumb and finger in children. J. Hand Surg..

[35] Lee Z.L., Chang C.H., Yang W.Y., Hung S.S., Shih C.H. (2006). Extension splint for trigger thumb in children. J. Pediatr. Orthop..

[36] Cardon L.J., Ezaki M., Carter P.R. (1999). Trigger finger in children. J. Hand Surg..

[37] Tordai P., Engkvist O. (1999). Trigger fingers in children. J. Hand Surg..

[38] Bae D.S., Sodha S., Waters P.M. (2007). Surgical treatment of the pediatric trigger finger. J. Hand Surg..

[39] Czarnecki P., Martinelli F. (2023). Lateral band snapping syndrome in the little finger—Easy to misdiagnose, easy to mistreat. Case report. Chir. Narządów Ruchu Ortop. Pol..

[40] Kapadia K., Shah S., Galvez M.G. (2022). Pediatric Wide-Awake Local Anesthesia No-Tourniquet Hand Surgery: A Practical Approach. J. Hand Surg. Glob. Online.

[41] Senrui H. (1984). Congenital clasped thumb combined with Waardenburg syndrome in three generations of one family: An undescribed congenital anomalies complex. J. Pediatr. Orthop..

[42] Flatt A.E. (1994). The Care of Congenital Hand Anomalies.

[43] Crawford H., Horton C., Adamson J. (1966). Congenital aplasia or hypoplasia of thumb and fingers extensor tendons. J. Bone Jt. Surg..

[44] Serbest S., Tosun H.B., Tiftikci U., Gumustas S.A., Uludag A. (2015). Congenital Clasped Thumb That Is Forgetten a Syndrome in Clinical Practice: A Case Report. Medicine.

